# Advances in Extremophile Research: Biotechnological Applications through Isolation and Identification Techniques

**DOI:** 10.3390/life14091205

**Published:** 2024-09-23

**Authors:** Giovanni Gallo, Martina Aulitto

**Affiliations:** 1 Division of Microbiology, Faculty of Biology, Ludwig-Maximilians-Universität München, 82152 Martinsried, Germany; giovanni.gallo@lmu.de; 2 Department of Biology, University of Napoli Federico II, Complesso Universitario Monte Sant’Angelo, 80126 Napoli, Italy

**Keywords:** extremophiles, isolation techniques, astrobiology, extremozymes, biotechnology

## Abstract

Extremophiles, organisms thriving in extreme environments such as hot springs, deep-sea hydrothermal vents, and hypersaline ecosystems, have garnered significant attention due to their remarkable adaptability and biotechnological potential. This review presents recent advancements in isolating and characterizing extremophiles, highlighting their applications in enzyme production, bioplastics, environmental management, and space exploration. The unique biological mechanisms of extremophiles offer valuable insights into life’s resilience and potential uses in industry and astrobiology.

## 1. Introduction

Extremophiles are microorganisms that inhabit a wide range of ecological niches across the globe, from polar regions and underground mines to oil fields and even the stratosphere [1]. They play a vital role in energy flux and biogeochemical cycles, with a particularly dominant presence in the world’s oceans, where their collective biomass surpasses that of multicellular marine organisms [2,3]. These microorganisms, which have existed for billions of years, represent a vast reservoir of genetic diversity with significant potential for applications in medicine and biotechnology. The study of extremophiles—found in extreme environments such as hot springs, deep-sea hydrothermal vents, polar ice, hypersaline lakes, and radiation-contaminated sites—has greatly enhanced our understanding of life’s resilience and adaptability [4]. The earth’s extreme environments challenge microorganisms to adapt through a range of sophisticated biochemical mechanisms, reflecting their unique ability to thrive under harsh conditions. Extremophiles, such as thermophiles and psychrophiles, showcase remarkable adaptability through distinct structural and molecular strategies. Thermophiles, which survive at high temperatures, exhibit enhanced protein stability primarily through increased hydrophobic interactions, salt bridges, and more disulfide bonds, which collectively contribute to their protein thermostability [5]. Structural studies reveal that these organisms often have shorter loops, more compact structures, and a higher proportion of charged and aromatic amino acids that stabilize their proteins at elevated temperatures. In contrast, psychrophiles adapt to cold environments by maintaining protein flexibility and increased entropy, facilitated by smaller, less bulky amino acid residues like glycine. Their proteins often feature more unsaturated fatty acids and fewer salt bridges, which enhance their functionality at lower temperatures. Genomic adaptations also play a crucial role, with thermophiles displaying higher G + C content in their tRNA and DNA, contributing to the stability of RNA structures and enhancing their thermal resilience. Psychrophiles, on the other hand, have a higher tRNA content and exhibit structural adaptations that protect against cold-induced destabilization. These mechanisms underline the diverse strategies extremophiles employ to maintain stability and functionality across extreme temperatures, offering valuable insights for biotechnological applications and understanding evolutionary processes [5]. Exploiting the complex taxonomy of prokaryotes is essential for gaining insights into evolution, not only for investigating the conditions that define habitability on Earth but also for providing critical insights into astrobiology, suggesting how life might exist beyond our planet [6]. Extremophiles, by thriving under extreme conditions, help define the environmental thresholds that support life, offering valuable information on adaptive responses and survival strategies. This review explores the diverse sources of extremophiles, their ecological significance, and the advancements in sampling and isolation techniques that have revealed their remarkable diversity. It also highlights their emerging applications in biotechnology, including their potential use in bioremediation and as sources of novel biomolecules in pharmaceutical and industrial processes [7]. From the boiling waters of geothermal pools to the icy expanses of polar regions, extremophiles offer a window into the early conditions of life on Earth and promise new avenues for technological innovation as research in this field continues to advance [8].

## 2. Source of Extremophiles

### 2.1. Hot Springs and Geothermal Areas

**Figure 1:**
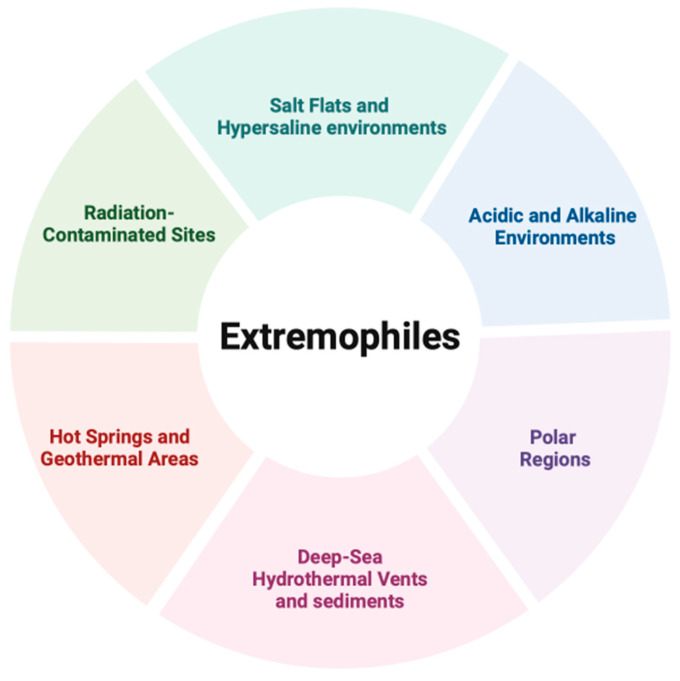
Schematic overview of the main environments where extremophilic microorganisms are found.

The hot springs scattered over our planet are extremely rich in microorganisms (Figure 1). Because of their extreme chemical and physical conditions, they are regarded as an open history book that allows us to study life at the beginning of our planet’s history and also gives us clues as to how life may be found on planets with more extreme climatic conditions [6,9,10]. Resembling the primitive conditions of early Earth, these hot springs have become pivotal research subjects, offering insights into ancient life forms [11]. Thermophiles like *Thermus aquaticus*, *Pyrococcus furiosus*, and *Aquifex aeolicus* thrive here, offering insights into life’s origins and temperature limits, and are important in astrobiology and biotechnology [12]. Initial explorations, primarily reliant on culture-based methods, have evolved substantially with advancements in molecular biology techniques. Culture-independent methods like 16S rRNA sequencing and metagenomics have greatly enhanced our understanding of hot-spring microbiota by identifying organisms that are hard to culture. However, they provide limited insights into microbial metabolism, physiology, and active community members and often rely on incomplete databases. In contrast, cultivation-dependent methods remain crucial for exploring microbial functions, metabolic pathways, and environmental interactions under controlled conditions. While sequencing offers broad data, cultivation provides essential insights into the physiological and ecological roles of microbes, offering a more complete picture of hot-spring ecosystems [9]. Furthermore, the study of thermophilic microorganisms provides important clues to potential extraterrestrial life. Terrestrial extremophiles, such as those found in hot springs or volcanic areas, thrive in environments that would have been considered uninhabitable just a few decades ago. These organisms are helping us redefine life’s limits and provide valuable models for studying the possibility of life on other planets with extreme conditions, such as Mars or the icy moons of Jupiter. For example, thermophilic bacteria found in hot springs in Yellowstone National Park are being studied as analogs for potential life forms on Europa, a moon of Jupiter where hydrothermal activity is thought to exist beneath the icy crust [11,12].

Moreover, these robust microorganisms also produce biomolecules and thermozymes suitable for various industrial applications, such as DNA polymerases from *T. aquaticus*, offering advantages in challenging industrial conditions [1]. Enzymes from thermophilic microbes are utilized in multiple industries, and the microbial communities possess enzymes capable of hydrocarbon degradation, indicating a potential for bioremediation in oil spill scenarios [13,14,15,16]. 

### 2.2. Deep-Sea Hydrothermal Vents and Sediments

The deep sea, covering most of Earth’s surface and largely unexplored, is an extreme environment characterized by adverse conditions such as low temperatures, high hydrostatic pressure, and no sunlight [17,18] (Figure 1). Despite these challenges, it is home to extremophiles that have remarkably adapted to survive in varying temperatures, pressures, pH levels, and salinity. These organisms are of significant scientific interest because of their unique ability to produce stable and active enzymes under extreme conditions [19]. The exploration of the deep sea has unveiled numerous unknown habitats and ecosystems, along with thousands of species, emphasizing its status as the largest biome globally, representing over 65% of Earth’s surface and more than 95% of the global biosphere [18]. These ecosystems are primarily populated by chemolithotrophic bacteria, such as *Methanopyrus kandleri*, which utilize inorganic compounds like hydrogen sulphide for energy [20]. Additionally, archaea like *Thermococcus gammatolerans* are prominent, adapting to extreme conditions [21]. Recent advancements in deep-sea extremophile research, breakthroughs in sampling techniques, and the rapid progress of molecular and omics technologies have spurred the development of novel industrial processes based on extremozymes. However, despite technological advances, only 5% of the deep oceans have been explored in detail, with less than 0.001% sampled and described in terms of biodiversity [22]. Recognized as a highly dynamic geo- and biosphere, the deep-sea environment, encompassing waters and sediments beneath 200 m depth, hosts diverse habitats such as canyons, seamounts, ridges, coral reefs, cold seeps, mud volcanoes, and more. Improved techniques for observing, mapping, and sampling the seabed have facilitated the exploration of these ecosystems [19]. Deep-sea microbial ecology, a relatively new branch of Ecology and Microbiology, has seen significant progress in the last two decades. Life has been documented throughout the deep sea, from the deepest trenches with active metabolic processes to sediments at 10,000 m depth and 1000 m below the seafloor. In particular, microbes in the deepest ocean trenches and those in sediments at different depths exhibit distinct metabolic adaptations due to varying environmental conditions. In the deepest trenches, such as the Mariana Trench, extreme pressures, low temperatures, and limited nutrients influence microbial metabolism [23]. These microbes often rely on piezophilic (pressure-loving) adaptations, using specialized enzymes that can function under high pressure and low energy availability. Many of them perform anaerobic respiration or fermentation, breaking down organic matter with minimal oxygen. Some microbes also utilize alternative electron acceptors, such as nitrate, sulfate, or carbon dioxide, for energy production. In contrast, microbes in sediments at 10,000 m depth, or 1000 m below the seafloor, face different challenges. At these depths, metabolic activity can be driven by buried organic matter and long-term geological processes, such as methane oxidation or sulfate reduction. These environments often exhibit more stable conditions, allowing microbes to thrive on chemolithoautotrophy—deriving energy from inorganic compounds like hydrogen or sulfur compounds—rather than relying solely on organic material [24]. Recent technological advancements in metagenomics have provided new perspectives for studying microbial life in deep-sea ecosystems [25]. Next-generation sequencing technology enables the comparison of microbial assemblages from different habitats, offering insights into their metabolic potential. The growing interest in the scientific community and technological improvements in exploring the dark portion of the oceans indicate a promising future for understanding microbial life in the most remote ecosystems globally [17].

### 2.3. Polar Regions

Recent studies of marine microbial communities have revealed complex biogeographical patterns, challenging the long-held notion of a uniform microbial seascape (Figure 1). Research has shown that variations in free-living marine microbial communities are closely linked to environmental factors such as salinity, depth, and oceanic fronts [26,27]. Several environmental factors influence the variation in free-living marine microbial communities in polar regions. Temperature is critical, as extreme cold favors psychrophilic microbes, while seasonal temperature changes drive shifts in microbial populations. Salinity fluctuations caused by sea-ice melting and formation create dynamic environments for microbes. Light availability, driven by polar day and night cycles, affects photosynthetic and heterotrophic microbes. Nutrient levels, sea-ice dynamics, and ocean currents further shape these communities alongside ocean acidification, which impacts pH-sensitive organisms. Together, these factors contribute to the rich microbial diversity found in polar ecosystems [28,29]. Polar regions, once thought to be desolate, have now been shown to be teeming with life, with distinctive ecosystems in permafrost, glaciers, and polar seas. Several psychrophilic bacteria have been isolated in these extreme environments, each with unique adaptations that allow them to survive and function at sub-zero temperatures. For example, *Psychrobacter* sp. and *Arthrobacter* sp. are commonly found in Antarctic soils and glaciers [30]. *Psychrobacter* sp., particularly strain G isolated from Antarctic environments, is known to produce cold-adapted enzymes with potential applications in industrial processes requiring low-temperature conditions. Similarly, *Arthrobacter* sp., which thrives in freeze–thaw cycles, produces carotenoids and other protective compounds that may have biotechnological applications. Another notable example is *Methanococcoides burtonii*, an archaeon isolated from Antarctic lakes, which exhibits unique cold-adaptation mechanisms such as the production of specialized proteins and enzymes [31,32,33]. These biochemical processes are of great interest in biosurfactant production, extracellular polymeric substance (EPS) synthesis, antibiotics, and enzyme production. The potential of these extremophiles extends beyond Earth, as astrobiologists are fascinated by the organisms found in sea ice, which may offer clues for life in the ice-covered seas of Jupiter’s moons and Mars. Furthermore, the escalating impact of climate change on sea-ice extent is a growing concern, particularly for key organisms like krill, which play a pivotal role in the Antarctic food chain. The study of these polar extremophiles not only enhances our understanding of life in extreme environments but also holds promising implications for biotechnology and the search for extraterrestrial life [34]. 

### 2.4. Salt Flats and Hypersaline Environments

Microorganisms in saline environments, spanning all three domains of life, have evolved to thrive in high salt concentrations (Figure 1). They are classified into weak (1–3% NaCl), moderate (3–15% NaCl), and extreme halophiles (15–30% NaCl) based on their salt tolerance [32,35]. These extremophiles, often termed polyextremophiles, can withstand high salinity and extreme pH and temperature conditions. In coastal hypersaline environments, higher salinity correlates with lower microbial diversity but increased numbers of extreme halophiles, particularly halophilic archaea. These latter primarily belong to the phylum *Euryarchaeota*, specifically the class *Halobacteria*, as various studies indicate. These organisms typically function as aerobic chemoorganoheterotrophs, although some species display metabolic versatility by utilizing alternative compounds such as NO_3_^−^, NO_2_^−^, ClO_3_^−^, or ClO_4_^−^ as final electron acceptors in anaerobic respiration. This metabolic diversity underscores their adaptability to fluctuating environmental conditions. Additionally, most halophilic archaea’s characteristic orange and red pigmentation arises from carotenoids alongside colored membrane proteins like bacteriorhodopsin, halorhodopsin, and other retinal proteins [36]. Conversely, bacterial abundance decreases along the salt gradient, peaking in areas with lower salinity. Molecular techniques like 16S rRNA gene analysis and in situ fluorescence hybridization have transformed our understanding of hypersaline environments, revealing the coexistence of bacteria and archaea contrary to earlier assumptions [37]. 16S rRNA gene analysis and in situ fluorescence hybridization (FISH) have significantly advanced our understanding of bacteria and archaea in hypersaline environments [38]. 16S rRNA gene analysis enables the identification and classification of diverse microbial species by sequencing a conserved gene, helping to reveal previously unknown organisms and their evolutionary relationships. This technique also provides valuable information on microbial community composition and potential metabolic functions. Meanwhile, FISH allows for direct visualization and differentiation of microorganisms within their natural habitat using fluorescent probes. This method reveals the spatial distribution of microbial populations, their interactions, and their relative abundance [38]. Together, these techniques illuminate how bacteria and archaea coexist and adapt in hypersaline conditions, uncovering their roles in biogeochemical cycles and their specific survival strategies in extreme environments. Archaea typically dominate these environments, comprising 70–95% of the microbial community, while bacteria represent 5–30% [39]. *Salinibacter ruber* is among the most common halophilic bacteria identified, alongside genera like *Salisaeta*, *Salicola*, *Halomonas*, and *Chromohalobacter*, albeit in smaller numbers [39,40,41]. Viruses become more prevalent in crystallizers, regulating archaeal populations. Subsequent sections will detail the most prevalent halophilic microbial species in salt-producing coastal and inland solar evaporation systems. Nearly 100 viruses prey on halophilic microorganisms, with 90 targeting haloarchaea and the remaining 10 infecting bacteria or eukaryotes [42]. These viruses are classified based on their morphology into various families: myoviruses, siphoviruses, pleomorphic viruses, podoviruses, and lemon-shaped viruses [43]. They feature contractile (myoviruses) or non-contractile (siphoviruses and podoviruses) tails and possess linear double-stranded DNA [44]. Regarding bacteria, fewer than ten viruses have been identified that target genera like *Pseudomonas*, *Halomonas*, *Salinivibrio*, *Salisaeta*, and *Salicola* [37]. 

### 2.5. Acidic and Alkaline Environments

Extremophiles are frequently encountered in environments with extreme pH levels, such as those characterized by very low acidity (pH 0.5–4) or high alkalinity (pH 9–12), classified as acidophiles and alkaliphiles [45] (Figure 1). Acidophiles represent a significant group of microorganisms with both ecological and economic importance, thriving in acidic environments found naturally in places like solfataric fields and sulfuric pools and in man-made settings such as areas associated with industrial activities like coal and metal-ore mining. This diverse assemblage comprises organisms from archaea, bacteria, fungi, algae, and protozoa, all adept at thriving in acidic conditions. They are distributed across various natural habitats, including solfataric fields, sulfuric pools, and geysers and in artificial environments influenced by human activities such as mining. Certain microbes, such as those belonging to the genera *Acidithiobacillus* and *Sulfolobus*, can utilize specialized sulfur oxidation pathways to generate energy. They oxidize reduced sulfur compounds (such as elemental sulfur, thiosulfate, or sulfide) to sulfuric acid (H_2_SO_4_), which not only provides them with energy but also contributes to the further acidification of their environment. The sulfur-oxidizing enzyme systems, like sulfur oxygenase reductase (SOR) in *Sulfolobus* and the Sox pathway in *Acidithiobacillus*, are essential to this process [46].

In 1956, Koki Horikoshi reported the discovery of the first alkali-tolerant eubacteria, *Bacillus circulans*, thriving in a culture flask with a pH 9 medium, emitting an ammonia-like smell [47]. Over the subsequent four decades, he extensively studied these organisms’ ability to survive in highly basic conditions, mostly around pH 10, while maintaining intracellular environments at pH 7–8 [45]. Aerobic alkaliphiles often coexist with neutrophilic microorganisms. Various aerobic alkaliphilic microorganisms, including *Bacillus*, *Micrococcus*, *Pseudomonas*, *Bogoriella*, *Halomonas*, *Alkalibacillus*, and eukaryotes such as yeast and fungi, have been isolated from different alkaline habitats such as soda lakes in Africa. Additionally, species like *Dietzia*, *Vagococcus*, *Paenibacillus*, *Marinobacter*, *Roseinatrobacter*, *Alkalimonas*, and *Rhodobaca* have been found in the alkaline Lonar Lake in India [48]. *Natronomonas pharaonis* and *Bacillus alcalophilus* are noteworthy among alkaliphiles, thriving in highly alkaline environments like soda lakes and alkaline soils. *Natronomonas pharaonis* can withstand high pH levels and salt concentrations, while *Bacillus alcalophilus* produces enzymes tailored for alkaline conditions, rendering it valuable in various industrial processes [49]. These extremophiles vividly illustrate the extraordinary adaptability of life forms to extreme environmental conditions.

### 2.6. Radiation-Contaminated Sites

Extremophiles found in radiation-rich environments, such as radioactive waste sites or regions exposed to intense cosmic radiation, have evolved remarkable adaptations to endure and even utilize radiation [50] (Figure 1). To withstand the indirect effects of radiation, microorganisms must efficiently scavenge and neutralize reactive oxygen species (ROS). As a result, radioresistant extremophiles have developed unique mechanisms to repair DNA damage caused by ionizing radiation. They often produce protective compounds like antioxidants to mitigate the harmful effects of radiation-induced oxidative stress [50]. Furthermore, these extremophiles have evolved proteins like superoxide dismutase and generate low molecular weight compounds such as glutathione, ascorbate, and trehalose, which act as natural scavengers of ROS [51]. Some extremophiles can even utilize radiation as an energy source through processes like chemosynthesis or radiotrophy, directly extracting energy from radiation or utilizing radiation-induced chemical reactions to power metabolic processes [52]. A notable example is the bacterium *Candidatus Desulforudis audaxviator*, discovered deep in South Africa’s Mponeng gold mine [53]. This microorganism is remarkable for surviving without sunlight, relying instead on the energy generated by water radiolysis [54]. The discovery of such a self-sustaining ecosystem has expanded the potential for finding life in non-illuminated environments beyond Earth, particularly on icy moons like Europa, where similar conditions might exist. Another example of extremophiles thriving in radiation-rich environments include certain bacteria like *Deinococcus radiodurans*. First isolated from radiation-sterilized meat containers in 1956, closely related isolates were found in high-level radioactive waste plumes at Hanford. Its capacity to endure ionizing conditions is credited to the production of protective antioxidants such as carotenoid deinoxanthin, along with biosorbent molecules utilized in treating heavy metal and radionuclide pollution [55].

## 3. Advancements in Sampling and Isolation Techniques for Extremophiles

Understanding extremophiles is crucial for advancing biosystems and bioprocesses and for gaining insights into their roles in global material cycles and ecosystem responses to human activity. Sampling extremophiles requires specialized techniques tailored to their extreme environments. For deep-sea exploration, remotely operated vehicles (ROVs) or submersibles are used, while heat-resistant containers are essential for environments like hot springs [56]. In polar regions, ice cores are collected to study psychrophiles, and pH-resistant materials are employed for environments with extreme acidity or alkalinity. Isolation and characterization are key to investigating their phylogenetic diversity and biogeochemical functions. Despite standardized protocols for isolation and cultivation [57], many extremophiles remain challenging to culture in laboratory settings, hindering the full exploitation of their biotechnological potential [58]. This is due to their highly specific and extreme environmental conditions, such as high temperatures, extreme pH levels, or high salinity, which are difficult to replicate accurately in a laboratory. Even slight variations from their native environment can result in poor growth or failure to culture. Additionally, extremophiles often have complex metabolic requirements and may depend on interactions with other microorganisms or specific chemical compounds in their natural habitats, which are difficult to simulate in isolation. This makes it challenging to cultivate them in pure cultures or under controlled lab conditions [11]. 

Molecular biology has revolutionized microbial community studies by enabling the analysis of unculturable microorganisms that traditional methods could not access. High-throughput sequencing offers a comprehensive genomic view of microbial diversity, but obtaining pure cultures of these microorganisms remains challenging, limiting their practical applications. To overcome these challenges, innovative techniques have been developed. In 2002, Kaeberlein’s team developed the “diffusion chamber” for analyzing microbial composition in marine sediments, which evolved into the ichip in 2010. The ichip places environmental cells into miniature diffusion chambers that mimic natural habitats, significantly increasing colony counts compared to synthetic media. This method has been especially effective for isolating soil and sediment microbes, revealing species of significant phylogenetic novelty. In a 2023 study by Juntian Zhao, the ichip was modified with gellan gum instead of agar, because it does not melt in the high temperatures of hot-spring waters, thus providing a stable support for microorganisms under these extreme conditions [59]. Additionally, the ichip was further improved by removing the upper and bottom plates, to facilitate the contact between the hot-spring water and the microorganisms. This adjustment, coupled with an increase in the pore diameter of the membrane, allows for more effective interaction and isolation of a broader spectrum of thermo-tolerant microbes. Notably, new thermo-tolerant species belonging to *Agromyces*, *Alkalihalobacillus*, and *Lysobacter* were identified, showcasing the ichip’s potential for cultivating previously unculturable microorganisms [59]. These developments provide valuable insights and resources for advancing extremophile research.

## 4. Emergent Biotechnological Applications

### 4.1. Pharmaceuticals

Extremophiles, organisms that thrive in extreme environments, are revolutionizing pharmaceutical biotechnology through the production of robust biomolecules, including enzymes known as extremozymes [4,60]. These enzymes, which can function under conditions that denature most other enzymes, such as extreme temperatures, high pH, and salinity, are ideal for industrial processes such as demanding drug synthesis and bioethanol production, where their ability to break down starch at high temperatures improves process efficiency and reduces energy costs. Research into the secondary metabolites produced by extremophiles has revealed substances with antibacterial, antitumor, and antiviral activity, opening up new avenues for the development of innovative medicines. For example, *Halobacterium salinarum*, an extreme halophile, has been studied for its ability to produce stable proteins in high-salinity environments, offering promising applications in drug formulation and marine biotechnology [61]. Similarly, *Sulfolobus acidocaldarius*, both an acidophile and thermophile, produces enzymes that are stable at low pH and high temperatures, making them suitable for drug synthesis and chemical degradation in industrial settings [62]. *Thermococcus kodakarensis*, another extremophile, produces KOD polymerase, an enzyme with high fidelity and precision in DNA replication, critical for molecular diagnostics [63] (Table 1). Bioremediation using extremophiles also offers strategies for degrading contaminants at contaminated sites that can be used to purify substrates used in pharmaceutical production. Advances in genomic and proteomic technologies have improved our understanding of these microorganisms, allowing us to explore new pharmaceutical applications. In addition, advances in synthetic biology have expanded the potential of extremophiles, with genetic engineering enhancing their ability to produce bioplastics and biofuels under extreme conditions. For example, the thermophilic bacterium *Geobacillus thermoglucosidasius* has been engineered to produce ethanol from biomass at high temperatures, allowing more efficient biofuel production by enabling simultaneous saccharification and fermentation processes [64]. Similarly, halophiles such as *Halomonas* species have been genetically engineered to produce polyhydroxyalkanoates (PHAs), a type of bioplastic, in high-salt environments, significantly increasing PHA yields. These modifications optimize production processes and contribute to making industrial production more sustainable [65]. In addition, the use of extremophiles to develop innovative solutions that improve the efficacy, stability, and safety of medicines is helping to advance modern medicine and support sustainability in industrial practices.

### 4.2. Bioremediation

Extremophiles have garnered significant attention for their unique capabilities in bioremediation, owing to their ability to thrive in conditions that are inhospitable to most other organisms. These microorganisms, including thermophiles, psychrophiles, halophiles, and acidophiles, have evolved unique enzymatic adaptations—extremozymes—that enable them to effectively metabolize and neutralize pollutants under extreme conditions. These extremozymes are tailored to remain functional and stable within the specific environmental extremes of temperature, pH, and salinity, facilitating the biodegradation of otherwise persistent pollutants [66]. Metal-tolerant extremophiles have demonstrated significant potential for bioremediation in mining-impacted environments. They are effective in reducing and precipitating toxic metals while also breaking down complex organic pollutants [67,68]. One example is the thermoacidophilic microorganisms, capable of surviving at high temperatures and low pH, which are beneficial for degrading hydrocarbons found in oil spills, thus accelerating bioremediation processes in marine and terrestrial ecosystems. Using these microorganisms can significantly decrease the time required for environmental cleanup and reduce reliance on harsh chemical treatments. A comprehensive overview of extremophiles involved in bioremediation is provided in several reviews. Among which, *Thermus aquaticus* and *Geobacillus stearothermophilus* have been reported to degrade organic pollutants in high-temperature environments, making them crucial for treating industrial waste. *Halomonas* and *Halobacterium* species are employed to manage waste in saline environments, essential for handling by-products from industries such as oil extraction. Notably, *Halomonas smyrnensis* has shown the ability to reduce hexavalent chromium in saline conditions, offering a promising method for treating heavy-metal-rich industrial effluents [69] (Table 1). *Ferroplasma acidarmanus* and *Acidithiobacillus ferrooxidans* are effective in addressing metal contaminants in acidic environments typically encountered in mining. Meanwhile, *Alcaligenes* and *Bacillus alcalophilus* are used to treat pollutants in alkaline conditions common in various industrial processes. Additionally, *Pseudomonas psychrophila* and *Psychrobacter* species are valuable for bioremediation in cold environments. These examples underscore the diverse capabilities of extremophiles in addressing pollution across a wide range of environmental conditions [6,70,71].

The enzymatic arsenal of extremophiles, notably extremozymes, is another critical aspect of their bioremediation potential. Enzymes such as thermoamilase can degrade starch-based pollutants at elevated temperatures, enhancing the efficiency of wastewater treatment in industries [72,73]. Psychrophilic enzymes from organisms like *Pseudoalteromonas* sp. have been shown to degrade pharmaceutical contaminants such as naproxen at low temperatures, making them invaluable for bioremediation in cold environments. The ability of extremophiles to function in extreme conditions, such as those found in radiation-contaminated sites, adds another layer of utility. Organisms like *Deinococcus radiodurans* can withstand high levels of ionizing radiation, using unique DNA repair mechanisms to survive and potentially degrade radioactive waste products [55] (Table 1). Acidophiles, like species of the genus *Acidithiobacillus*, demonstrate their unique biotechnological prowess in heavy-metal recovery from industrial wastes, leveraging their robust metabolic capabilities [74]. These microorganisms can endure highly acidic environments, facilitating the bioleaching process where heavy metals such as copper and uranium are efficiently extracted. Utilizing organic and inorganic acids, these organisms dissolve metals from waste materials, offering an eco-friendly alternative to conventional chemical-based metal recovery methods. The application of acidophiles extends beyond mere extraction, impacting environmental cleanup endeavors. Research into the genetic engineering of these acidophiles suggests a future where their capabilities can be enhanced, leading to more effective bioremediation strategies in various industrially polluted environments. Furthermore, the integration of acidophiles into bioremediation processes has been shown to not only recover valuable metals but also to contribute to the detoxification of contaminated sites, thus aligning with sustainable environmental management practices [75]. This capacity positions extremophiles as pivotal agents in the quest for eco-friendly and effective bioremediation strategies. Their deployment not only addresses the immediate need for pollutant degradation but also contributes to the long-term sustainability of environmental management practices.

### 4.3. Astrobiology

Extremophiles have emerged as vital models for understanding the potential for life beyond Earth, significantly impacting the field of astrobiology [11,12]. These organisms thrive in the harshest conditions on Earth, such as extreme temperatures, radiation, salinity, and acidity, offering insights into the types of environments that might support life elsewhere in the universe [76]. For instance, psychrophiles, adapted to cold environments, provide valuable insights into the possibilities for life on icy moons like Europa and Enceladus, where similar frigid conditions exist [2]. Extremophiles such as *Deinococcus radiodurans*, renowned for their resistance to high radiation levels, are studied for their potential to survive on planets with thin atmospheres, such as Mars, where radiation exposure is significant [77] (Table 1). Other extremophiles such as *Thermococcus gammatolerans* and *Pyrococcus furiosus* are notable for their survival in extreme temperatures, with the former enduring high radiation and the latter thriving in high heat, suggesting how life might withstand the intense conditions found on other planetary surfaces [21,78]. Interestingly, other examples are *Bacillus subtilis*, which exhibits resilience to desiccation and UV radiation, which is pertinent for understanding survival in the vacuum of space, and *Halobacterium salinarum*, which thrives in highly saline environments, similar to those found on other planets or moons with salty conditions [79]. Lastly, *Cryococcus glacialis*, a psychrophilic bacterium, endures freezing temperatures, offering insights into how life might persist in the icy realms of outer space. These extremophiles enhance our understanding of the limits of life and inform the search for extraterrestrial life by simulating the harsh conditions that might be encountered beyond Earth [80].

The biochemical innovations exhibited by extremophiles, including unique metabolic pathways and protective mechanisms against environmental stresses, provide a framework for hypothesizing extraterrestrial life forms [56]. For example, the discovery of extremophiles that utilize chemosynthesis in deep-sea hydrothermal vents suggests that similar life forms could exist on celestial bodies with subsurface oceans, where sunlight is absent but chemical energy sources are abundant. Additionally, extremophiles contribute to astrobiology by challenging our understanding of life’s adaptability [81]. Their ability to endure multiple stressors simultaneously, such as high radiation and desiccation, expands the traditional definition of habitable zones and influences the design of life-detection experiments for space missions. The study of these organisms not only aids in identifying biosignatures that may be detected by future Mars rovers and other space probes but also enhances our understanding of potential survival strategies for life in the extreme conditions of outer space. As technology advances, the exploration of extremophiles through omics technologies continues to unlock new possibilities for astrobiology [12]. The integration of genomics and metagenomics has led to a deeper understanding of the genetic and metabolic potential of extremophiles, providing critical insights into how life might adapt and thrive on other planets. Overall, extremophiles are reshaping our understanding of life’s limits and pushing the boundaries of astrobiological research, making them indispensable in the search for life beyond Earth.

### 4.4. Materials Science

Material science is a multidisciplinary field that explores the properties, processing, and applications of materials to develop new technologies and improve existing ones. It plays a critical role in advancing industries ranging from aerospace and automotive to healthcare and electronics by providing the foundation for creating stronger, lighter, and more durable materials. With growing concerns over sustainability and environmental impact, there is an increasing demand for materials that are not only high-performing but also eco-friendly [82]. In this context, the use of extremophiles offers a novel and promising approach to developing advanced materials, particularly in the production of bioplastics and EPSs [83].

Traditional bioplastic production relies heavily on renewable carbon sources like sugars derived from crops such as corn, potato, or soy. However, this approach is costly, both in terms of feedstock and the energy-intensive processes required [84]. For instance, it takes approximately 4.3 kg of sugar to produce just 1 kg of bioplastic, which not only demands large-scale agricultural input but also increases the overall cost due to the need for sterile conditions and energy-intensive extraction protocols [84]. In contrast, extremophiles offer a more cost-effective and sustainable approach, in which their ability to thrive in extreme environments reduces the risk of contamination from other microorganisms. This makes the production process more stable and less reliant on stringent sterilization protocols, which can lower overall production costs [1]. Another key advantage of using extremophiles for bioplastic production is their ability to accumulate PHAs during their growth phase. Unlike traditional bioplastic production, which requires a two-phase process where cells are stress-induced during the late exponential growth phase, extremophiles naturally produce and accumulate PHAs as part of their regular metabolic activities. This not only simplifies the production process but also reduces the energy costs associated with nutrient limitations and stress induction. Despite their potential, the commercial exploitation of extremophiles for bioplastic production is still in its infancy. To date, only a few extremophiles have been commercialized for polymer production, while most research has focused on their use for producing extremozymes. However, the fact that extremophiles accumulate PHAs to protect their cellular structures from environmental stress opens new avenues for manipulating these organisms for commercial bioplastic production. In particular, PHAs in psychrophiles play a crucial role in protecting cells from oxidative stress and facilitating survival under microaerobic conditions. For example, *Pseudomonas* sp. 14–3 utilizes PHAs to shield itself from oxidative damage, while *Pseudomonas extremaustralis* sp. nov. relies on PHAs to endure low-oxygen environments. Moreover, several strains of halophilic archaea, including genera such as *Haloferax*, *Halobacterium*, *Haloarcula*, and *Haloquadratum*, have been found to accumulate PHAs from inexpensive carbon sources and industrial waste. Among these, *Haloferax mediterranei* [85] stands out as a highly promising and well-researched candidate for commercial PHA production, known for its efficiency and adaptability to various carbon sources. Its capabilities could be further enhanced through genetic modifications, a possibility that is likely to be realized in the near future [84].

Extremophiles, especially those capable of producing EPSs, are increasingly recognized as valuable resources in biotechnology and material science. EPSs, which are high-molecular-weight polymers composed of sugar residues, are notable for their structural complexity, natural origin, non-toxicity, biocompatibility, and biodegradability [86]. While EPSs derived from mesophilic microorganisms have been extensively studied and applied across various industries, extremophiles—organisms that thrive in extreme conditions—are drawing attention for their unique EPS production capabilities, which may result in biopolymers with superior properties. Interestingly, a novel EPS, named TA-1, was isolated from the biofilm of *Thermus aquaticus* YT-1 and characterized as a 500 kDa molecule composed of tetrasaccharide units of galactofuranose, galactopyranose, and N-acetylgalactosamine. Analysis revealed that TA-1 stimulates macrophages to produce cytokines TNF-α and IL-6 through Toll-like receptor 2 (TLR2). This immunomodulatory effect was confirmed by TA-1’s ability to induce IL-6 in wild-type mice macrophages but not in TLR2−/− mice. Moreover, TA-1’s activity was demonstrated to be involved in the MyD88/TIRAP pathway and suggests potential use as an adjuvant to enhance immune responses [87]. Another example of an EPS producer is the halophilic archaeon *Halobacterium salinarum* R1, known for its ability to form characteristic biofilm structures on surfaces under physiological conditions. Notably, in the study, Simoes et al. 2018 demonstrated how the EPS matrix in *H. salinarum* R1 biofilms contributes to enhanced resistance and changes in biofilm architecture under metal stress [88]. Furthermore, *Acidithiobacillus ferrooxidans* ATCC 23270T has been demonstrated to produce EPSs in Li et al. (2016), and it was also investigated how EPSs influence bacterial attachment to minerals [84,89]. 

In this context, the Bulgarian Academy of Sciences has been actively researching the rich biodiversity of Bulgaria’s thermal springs and saline environments, aiming to isolate novel thermophilic and halophilic microorganisms capable of producing EPSs with unique and valuable properties. For instance, the moderately halophilic bacterium *Chromohalobacter canadensis* has been identified as a prolific EPS producer, underscoring the potential of these extremophiles in various biotechnological applications. Recent explorations have uncovered promising thermophilic EPS producers and several from saline environments, revealing impressive taxonomic and bacterial diversity [90]. The EPS produced by *C. canadensis* has shown promising rheological properties, such as high viscosity and stability, making it suitable for applications in food, pharmaceuticals, and environmental protection. Compared to other halophilic bacteria, *C. canadensis* demonstrates a higher yield of EPS under saline conditions, offering a competitive edge in environments where other organisms might struggle to maintain high production levels. Additionally, the biochemical characteristics of its EPS, such as unique monosaccharide composition and potential bioactivity, can differ from those produced by other halophiles, potentially leading to novel applications. The robust nature of *C. canadensis*, coupled with its efficient EPS production in extreme environments, makes it a valuable organism for exploring new bioproducts and enhancing industrial processes that operate in saline or hypersaline conditions. Notable among these are four thermophilic species—*Geobacillus tepidamans* V264, *Aeribacillus pallidus* 418 [91], *Brevibacillus thermoruber* 423 [92], and *Brevibacillus thermoruber* 438 [93]—along with the halophilic strain *Chromohalobacter canadensis* 28 [90] (Table 1). These isolates are promising candidates for further research and development, offering significant potential for advancing the use of EPSs in a range of biotechnological applications.

Extremophiles present a compelling alternative for the production of bioplastics and other advanced materials due to their ability to thrive and produce valuable compounds under extreme conditions. While the commercial exploitation of extremophiles for material production is still in its early stages, their potential for revolutionizing bioplastic production and other biotechnological applications is immense. As research continues to uncover new extremophilic species and optimize their use, these remarkable organisms could play a key role in the future of material science, driving innovation in eco-friendly and high-performance materials. One of the primary obstacles is the difficulty in cultivating these organisms on a large scale. Maintaining the specific conditions required for their growth can be costly and technically demanding, often resulting in lower yields and higher production costs compared to conventional microorganisms. Another challenge lies in the genetic and metabolic complexity of extremophiles [94]. These organisms have unique metabolic pathways that are not yet fully understood, making it difficult to optimize their production processes for industrial applications. To overcome these challenges, advancements in biotechnology and synthetic biology are crucial. Improved genetic engineering techniques could enable the modification of extremophiles to optimize their growth and production capabilities under less extreme, more economically viable conditions. Enhanced understanding of the metabolic pathways in extremophiles through omics technologies would facilitate the identification and manipulation of key genes involved in material production, further improving yields and product consistency [94]. 

**Table 1:** **Main applications of extremophiles.** Only the microorganisms reported in the text are listed.

Biotech Application	Microorganism	Reference
Marine Biotechnology	*Halobacterium salinarum*	[61]
Stable enzyme	*Sulfolobus acidocaldarius*	[62]
KOD polymerase	*Thermococcus kodakarensis*	[63]
Bioremediation	*Halomonas smyrnensis*	[69]
Astrobiology and bioremediation	*Deinococcus radiodurans*	[55]
Astrobiology	*Thermococcus gammatolerans*	[69]
Astrobiology	*Pyrococcus furiosus*	[78]
Astrobiology	*Bacillus subtilis*	[79]
Astrobiology	*Halobacterium salinarum*	
Astrobiology	*Cryococcus glacialis*	[80]
PHAs	*Pseudomonas* sp. 14–3	[34]
PHAs	*Pseudomonas extremaustralis* sp.	[73]
PHAs	*Haloferax mediterranei*	[85]
EPS	*Methanococcoides burtonii*	[32]
EPS	*Geobacillus tepidamans* V264	[95]
EPS	*Aeribacillus pallidus* 418	[91]
EPS	*Brevibacillus thermoruber* 423	[92]
EPS	*Brevibacillus thermoruber* 438	[93]
EPS	*Chromohalobacter canadensis* 28	[90]
EPS	*Halobacterium salinarum* R1	[88]
EPS	*Acidithiobacillus ferrooxidans* ATCC 23270T	[74]
EPS	*Thermus aquaticus* YT-1	[88]

## 5. Conclusions

Extremophiles, thriving in some of the most inhospitable environments on Earth, offer profound insights into both the limits of life and its potential applications. These microorganisms exemplify nature’s remarkable adaptability, from geothermal hot springs and deep-sea hydrothermal vents to the polar regions and hypersaline environments. They provide a window into the conditions of early Earth and suggest the possible existence of life in similar extreme conditions elsewhere in the universe. 

Technological advancements in sampling and isolation techniques have expanded our ability to explore and cultivate these organisms, revealing various extremophiles with unique capabilities. These discoveries have spurred innovations in biotechnology, including the development of extremozymes that enhance industrial processes, the production of bioplastics from extremophiles, and novel bioremediation strategies for pollutant cleanup. In astrobiology, extremophiles offer crucial clues about the potential for life beyond Earth, guiding the search for biosignatures on other planets and moons. Their ability to endure extreme conditions informs our understanding of life’s resilience and adaptability, shaping future missions and experiments in space exploration.

Overall, extremophiles stand at the intersection of environmental science, biotechnology, and space exploration. Their review advances our scientific knowledge and drives practical applications that address critical challenges in industry and environmental management. As research continues to evolve, extremophiles are poised to make significant contributions to various fields, highlighting the boundless potential of life in extreme conditions and paving the way for future innovations.
